# West Nile virus neuroinvasive disease: neurological manifestations and prospective longitudinal outcomes

**DOI:** 10.1186/1471-2334-14-248

**Published:** 2014-05-09

**Authors:** John Hart, Gail Tillman, Michael A Kraut, Hsueh-Sheng Chiang, Jeremy F Strain, Yufeng Li, Amy G Agrawal, Penny Jester, John W Gnann, Richard J Whitley

**Affiliations:** 1 Center for BrainHealth, University of Texas at Dallas, Dallas, TX, USA; 2 The Johns Hopkins University School of Medicine, Baltimore, MD, USA; 3 NIH, Bethesda, MD, USA; 4 Medical University of South Carolina, Charleston, SC, USA; 5 University of Alabama at Birmingham, Birmingham, AL, USA

**Keywords:** West Nile virus, Encephalitis, Neurological deficit, 3MS, Coma

## Abstract

**Background:**

West Nile Virus (WNV) is a mosquito-borne flavivirus that has caused ongoing seasonal epidemics in the United States since 1999. It is estimated that ≤1% of WNV-infected patients will develop neuroinvasive disease (West Nile encephalitis and/or myelitis) that can result in debilitating morbidities and long-term sequelae. It is essential to collect longitudinal information about the recovery process and to characterize predicative factors that may assist in therapeutic decision-making in the future.

**Methods:**

We report a longitudinal study of the neurological outcomes (as measured by neurological examination, Glascow Coma Scale, and Modified Mini-Mental State Examination) for 55 subjects with WNV neuroinvasive disease (confirmed by positive CSF IgM) assessed on day 7, at discharge, and on days 14, 30, and 90. The neurological outcome measures were coma (presence and degree), global cognitive status, presence of cranial neuropathy, tremors and/or weakness.

**Results:**

At initial clinical presentation 93% presented with a significant neurological deficit (49% with weakness, 35% with tremor, and 16% with cranial neuropathy). The number of patients with a cognitive deficit fell from 25 at initial evaluation to 9 at their last evaluation. Cranial neuropathy was present in 9 at onset and in only 4 patients at study conclusion. Of the 19 patients who had a tremor at enrollment, 11 continued to exhibit a tremor at follow-up. Seven patients died after initial enrollment in the study, with 5 of those having presented in a coma. The factors that predict either severity or long-term recovery of neurological function include age (older individuals were weaker at follow-up examination), gender (males recovered better from coma), and presentation in a coma with cranial nerve deficits (had a poorer recovery particularly with regard to cognition).

**Conclusions:**

This study represents one of the largest clinical investigations providing prospectively-acquired neurological outcomes data among American patients with WNV central nervous system disease. The findings show that the factors that influence prognosis from the initial presentation include age, gender, and specific neurological deficits at onset.

**Trial registration:**

ClinicalTrials.gov identifier: NCT00138463 and NCT00069316.

## Background

West Nile Virus (WNV), a zoonotic arbovirus of the Flaviviridae family, is transmitted from infected birds to humans via mosquito vectors [1]. First identified in Uganda in 1937, numerous outbreaks of human WNV disease have been reported globally. The first cases in the United States appeared in 1999, initially occurring as a localized epidemic of encephalitis in New York City [1-3]. Subsequently, the WNV epidemic spread across the United States, peaking at 9862 reported cases with 264 documented deaths in 2003 (CDC statistics). WNV established a relatively stable pattern of endemic infection in the U.S., although there was a resurgence in 2012, with 5674 reported cases and 286 deaths (CDC statistics).

About 70-80% of individuals infected by WNV are asymptomatic and do not come to medical attention, while approximately 20-30% have “West Nile Fever” without evidence of neurologic involvement [4-7]. Neuroinvasive disease accounts for less than 1% of WNV cases, but can result in severe neurological sequelae and death [8]. The mortality rate is between 10% and 30% for patients with neuroinvasive WNV disease, or <0.1% of all infected patients [8,9].

The virus invades a wide range of anatomic structures of the central nervous system (CNS) [10-12]. Neuroinvasive WNV disease can manifest as one of three main clinical entities: West Nile meningitis (WNM), West Nile encephalitis (WNE), or acute flaccid paralysis (AFP) [13]. Myelitis with AFP can occur independently or in conjunction with WNE. CNS disease has been associated with changes in cognitive status, muscle weakness, motor neuron disease (flaccid paralysis and hyporeflexia), cranial nerve palsies (most commonly facial nerve palsy), Guillain-Barre like syndrome (GBS), or movement disorders (e.g., myoclonus, tremor, parkinsonism) [1,8,14-18]. This spectrum of clinical findings is mirrored by radiologic abnormalities that involve the basal ganglia, thalami, cerebellum, and brain stem [19-21]. Importantly, imaging studies may be normal or demonstrate only nonspecific changes in patients with acute neuroinvasive WNV infection and are thus of limited utility for diagnostic purposes [1,12].

Clinical signs and symptoms of disease during the acute phase of WNV infection have only modest prognostic value. The severity of acute symptoms is not an accurate predictor of duration or extent of recovery, which can be quite variable [4,8,10,22]. Older age (over 50 years) and medical co-morbidities have been associated with higher rate WNV-related morbidity and mortality [3,23]. The variability of the degree of resolution of neurologic disease is particularly striking. While some initial symptoms may remit to the premorbid baseline, others can persist for over a year after disease onset [22,24]. Most patients with WNM and no focal neurological deficits usually recovery fully, but there are frequent subjective complaints of prolonged fatigue and weakness [16,25]. Unlike WNM, patients diagnosed with WNE have a significant mortality rate (~20%) and those who survive are likely to experience persistent movement disorders (e.g., tremors, bradykinesia) for months to years [4]. AFP has the worst overall prognosis and a high mortality rate, which can reach 50% when neuromuscular respiratory failure occurs [4]. Severe residual muscle weakness is common, but improvements of muscle strength have been observed within the first 6 months to 1 year after disease onset, such that patients can regain partial function of the paralyzed extremities [4,10,22,26].

While these studies have provided useful insight into the initial neurological presentation of and subsequent recovery from WNV neuroinvasive disease, prospectively collected, longitudinal data defining the clinical course of WNV neuroinvasive disease have been limited. These types of studies have been difficult to perform due to the sporadic nature and unpredictable geographic distribution of the WNV outbreaks. We report here a longitudinal, prospective, multi-center evaluation of neurologic outcomes of patients with neuroinvasive WNV disease in the U.S.

## Methods

The current report is derived from a randomized, double-blinded, placebo-controlled clinical trial performed by the National Institute of Allergy and Infectious Diseases (NIAID) Collaborative Antiviral Study Group (CASG) to assess the safety and potential therapeutic utility of passive immunotherapy in patient with WNV neurologic disease. Patients were randomized to receive high-titer WNV immunoglobulin (Omr-IgG-am^TM^), intravenous immune globulin (Polygam® S/D), or normal saline at a ratio of 3:1:1.

This clinical trial was conducted between the Fall of 2003 and the Fall of 2006 at 43 sites in the U.S. and Canada and was approved by local institutional review boards (the CASG Central Unit IRB is as follows: the UAB Institutional Review Board for Human Use, 701 20th Street South, AB 407 Birmingham, AL 35294–0104; trial registration: NCT00138463 CASG 211 and NCT00069316 CASG 210). This report will focus on a detailed evaluation of neurologic presentations and outcomes while results related to the therapeutic interventions will be published separately.

### Enrollment criteria

Enrollment was limited to patients with suspected WNV disease based on clinical findings (with subsequent confirmatory diagnostic testing) or patients in whom WNV disease had already been proven. For purposes of this study, laboratory diagnosis of WNV infection was established by either a positive serum or cerebrospinal fluid (CSF) anti-WNV IgM serologic assay. WNV serologic testing for all subjects was performed at the Clinical Virology Laboratory at the University of Alabama at Birmingham using the Focus Technologies ELISA methodology. No additional confirmatory diagnostic testing was undertaken. The subjects provided written informed consent (or consent was provided by a legal representative). Subjects were enrolled based on one of two eligibility categories:

1. Hospitalized patients greater than age 18 with encephalitis and/or myelitis presenting as a new neurologic abnormality (asymmetric extremity weakness without sensory abnormality, other neurologic abnormality, or altered level of consciousness), plus fever within the previous 4 days; and CSF examination within the previous 96 hours demonstrating an absence of organisms by routine microbiologic testing (including polymerase chain reaction [PCR] test for herpes simplex virus), WBCs > =4 per mm^3^ (corrected for significant red blood cell contamination), and ratio of CSF: plasma glucose of ≥ 40% (CSF glucose/plasma glucose ≥ 0.4). The etiology of illness was subsequently confirmed to be caused by WNV by specific serologic testing, as above.

2. Hospitalized patients without encephalitis and/or myelitis who met the following criteria: a positive IgM serology for WNV (performed at the local site laboratory; subsequent confirmation at the study central laboratory required) in blood or cerebrospinal fluid, and clinical illness compatible with WNV infection as defined by occurrence of ≥ 3 of the following findings during the preceding ≤ 10 days (diarrhea; headache, fever > 38 ºC; nausea and/or vomiting; myalgias and/or arthralgias; nuchal rigidity; macular or papular rash, new neurological abnormality); and a risk factor for the development of WNV neurologic disease as defined by being older than 40 years of age, or being older than 18 and immunocompromised.

Exclusion criteria included: unable to provide informed consent; history of intolerance (including anaphylaxis) to intravenous immunoglobulin (IVIg) or related compounds; known history of IgA deficiency; known history of hypersensitivity to maltose; history of hyperviscosity syndrome; congestive heart failure as defined as Class III or IV by the New York Heart Association Classification; serum creatinine > 2.5 mg/dL or requirement of hemodialysis; another plausible explanation for clinical findings (such as structural brain lesion, cerebrovascular accident, or other infectious disease, including confirmed infections with other flaviviruses); pregnant or breastfeeding.

#### Study oversight

The clinical trial was supported by the National Institute of Allergy and Infectious Diseases under an Investigational New Drug Application that was held by the sponsoring Institution. All patients were clinically managed at the local participating institutions. All data were recorded on standardized case record forms. Data verification was performed by an independent monitor to guarantee accuracy and completeness.

Stopping rules were built into this study based on the number of observed serious adverse events. An independent Data Safety and Monitory Board (DSMB) reviewed all clinical data, including the neurologic assessments presented herein. With guidance from the DSMB, subject enrollment was terminated in 2006 due to: 1). slower-than-expected enrollment; 2). expiration of the Omr-IgG-am™ study product; and 3). inability to procure sufficient quantities of WNV IgG-free IVIg of U.S. origin to use as a control.

#### Study design

At the time of randomization, each of the participants underwent measurement of vital signs, a general physical examination and, and a detailed neurological evaluation which included determination of scores on the Glasgow Coma Scale (GCS) and the adapted Glasgow Outcome Score (GOS), the Modified Mini-Mental Status test (3MS), the Modified Rankin Scale (MRS), and the Barthel Index (BI). Safety laboratories and confirmatory CSF were obtained followed by initiation of study medication according to the randomization schedule. The above-listed assessments were then re-administered prior to infusion, on day 7, at discharge, and on days 14, 30, and 90. For the purposes of this report, the data related to the neurological findings, GCS, and 3MS are reported.

Each of the physicians who performed the neurological examination was trained by a lead neurologist (J.H.) with experience and expertise in training research study personnel. The training consisted of reviewing the neurological examination with the physicians in a group session, supervising their practicing and performance of the examination, supplying the physicians with detailed instructions on performing and scoring the neurological examination, and supplying an instructional video for reference. Refresher training sessions were held annually.

#### Neurological assessments

For the purposes of this report, the first and last available time points of testing for each individual were analyzed. Given the heterogeneity of the neurological findings, the results of the studies were categorized as follows:

1. The Glascow Coma Scale (GCS) was performed to determine the patient’s level of consciousness at entry. If the patient was in coma, as indicated by a GCS of 8 or less, the other measures could not be reliably assessed in most cases and these were noted as not testable (e.g., 3MS, strength, etc.).

2. The Modified Mini-Mental Status Test evaluated cognitive performance. A score of 78 or less out of 100 was categorized as impaired [27].

3. The presence of a cranial neuropathy was evaluated by assessing pupil size, pupillary response, visual fields to threat, extraocular movements, ptosis, smile for facial strength, gag reflex, and tongue deviation.

4. Tremors. The presence of tremors and the number of involved limbs and/or head were noted. Evaluators also recorded the presence of intention tremors vs. resting tremors.

5. Weakness. The number of limbs that were weak, the strength rating using the MRC 5 point strength scale, and muscle tone were documented. Reflexes were also recorded. The strength ratings typically noted were 0, 3, 4, or 5. Muscle tone and reflexes were correlated with observed limb strength to see if the typical upper motor neuron (UMN) or lower motor neuron (LMN) patterns were present.

To facilitate analysis of the dataset, the number of limbs that were weak was noted and the strength scores of all of the muscle groups (deltoid, biceps, triceps, brachioradialis, wrist extensors, wrist flexors, and grip for the upper extremity [UE]; hip flexors, quadriceps, hamstrings, foot dorsiflexors, and foot plantar flexors for the lower extremity [LE]) were averaged to provide a limb strength score.

#### Neurologic scoring

For each patient, a score was determined at enrollment and again on follow-up. With the exception of those consistently in a coma, the Outcome Score ranged from 0 (no cognitive deficits, no tremor, no cranial nerve problems, no weakness) to 6. One point was awarded for each deficit.

#### Biostatistical considerations

This was a randomized, placebo-controlled, multicenter trial designed to assess and characterize the safety and tolerability of Omr-IgG-am™ in a population of hospitalized patients with confirmed or suspected WNV disease. Confirmed patients were those who met protocol inclusion/exclusion criteria and had a positive WNV IgM serologic result from CSF and/or serum. All subjects included in this analysis are confirmed cases. The primary endpoint was to evaluate safety in the group receiving Omr-IgG-am™ versus the groups receiving conventional IVIg or placebo (normal saline), as defined by the number of serious adverse events. Secondary endpoints included pharmacokinetics of specific anti-WNV antibodies, mortality, and morbidity based on neurological evaluations, which are described above. A total of 100 patients was projected to receive investigational therapy with Omr-IgG-am™ (0.5 g/kg) or one of two controls: IVIG (Polygam® S/D) or normal saline at a 3:1:1 randomization ratio in order to achieve a reasonable estimation of the treatment related serious adverse event with 95% confidence intervals. However, this study was terminated early (for reasons discussed above) with a total of 64 subjects enrolled; 2 subjects were determined to be ineligible and received no study medication. For the purposes of this report, the data related to the neurological findings, GCS, and 3MS are reported to the subgroup of subjects (n = 55 of 62) confirmed to have WNV infection. WNV IgM was positive (serum and/or CSF) in 55/55; WNV IgG was positive in serum in 53/55 subjects. Both baseline (day 0) and the last follow up (day 90) neurological assessment were examined.

Data are presented using descriptive statistics. Normally-distributed variables will be reported as means with standard deviations; non-normally distributed continuous variables will be reported as medians with ranges; categorical data is reported as frequency and proportion. Cochran-Mantel-Haenszel Statistics or Fisher’s exact were used to exam the association for categorical variables. Logistic regression analysis and other linear regression analyses were used to evaluate associations between patients’ demographics and outcomes. These statistical analyses are exploratory. Statistical analysis is performed using SAS 9.3 software.

## Results

### Demographic characteristics

A total of 64 subjects were enrolled into a controlled, randomized study of WNV immune globulin therapy for patients with WNV neuroinvasive disease. Nine subjects were subsequently excluded for failing to meet study entry criteria. Subjects were enrolled at 24 sites; the largest numbers of subjects were recruited at Idaho Falls, ID (9), Fargo, ND (6), University of Colorado at Denver, CO (6), and the University of New Mexico, Albuquerque, NM (6). The first subject was enrolled on September 21, 2003 and the last subject enrolled on September 28, 2006.

The median age of the study population was 59 years (range 34–85 years) and did not vary significantly among treatment groups. The study population was 72.7% Caucasian, 3.6% Black, 18.2% Hispanic, 3.6% Native American, and 1.8% other. Subjects were 69.1% male and 30.9% female. The demographic characteristics for the three study populations are presented in Table 1.

**Table 1:** The demographic characteristics for the three study populations

	**Omr-IgG-AM WNV (N=33) (%)**	**POLYGAM WNV (N=11) (%)**	**SALINE WNV (N=11) (%)**	**Total WNV (N=55) (%)**
**Race**				
Caucasian/Not Hispanic	22 (66.7)	9 (81.8)	9 (81.8)	40 (72.7)
Black/Not Hispanic	1 (3.0)	-	1 (9.1)	2 (3.6)
Hispanic	8 (24.2)	1 (9.1)	1 (9.1)	10 (18.2)
Asian or Pacific Islander	-	-	-	-
American Indian or Native American	1 (3.0)	1 (9.1)	-	2 (3.6)
Other	1 (3.0)	-	-	1 (1.8)
**Gender**				
Male	23 (69.7)	7 (63.6)	8 (72.7)	38 (69.1)
Female	10 (30.3)	4 (36.4)	3 (27.3)	17 (30.9)
**Age in Year**				
Mean ± SE	58.00 ± 2.18	55.36 ± 4.26	62.18 ± 4.83	58.31 ± 1.82
Median	56	54	60	59
Min-Max	34 - 83	34 - 77	36 - 85	34 - 85

No subjects were terminated from the study due to non-fatal adverse events. Death (N = 7) was the most frequent reason for early study termination. For these 7 subjects, the last evaluation day was as follows: 3 were at day 7, 3 at day 14, and 1 at day 30.

### Treatment effects

Analyses of treatment effects from the clinical trial will be summarized only briefly to justify merger of treatment groups for this analysis; detailed analysis of the treatment effects will be published elsewhere.

The primary endpoint for the study was safety in the experimental drug arm versus each of the 2 control arms, as determined by the total number of serious adverse events (SAEs). The differences in frequency of SAEs among the 3 treatment groups were not statistically significant. Specifically, there were no differences in neurological SAEs among the 3 treatment groups.

As an indicator of therapeutic efficacy, a combined endpoint of mortality and morbidity was calculated among confirmed subjects, as assessed by 4 scoring systems (the Barthel Index, the MRS, the GOS, and the 3MS) at 3 months in the experimental treatment group versus 2 control groups. Each subject was placed into one of 4 functional categories, using pre-defined breakpoints: normal, mildly/moderately impaired, severely impaired, or dead. There were no statistically significant differences in the proportions of patients falling into the 4 functional categories for the 3 treatment cohorts. Outcomes were further dichotomized into “favorable” and “unfavorable” categories using predetermined cut-points. Exact 95% confidence intervals on the proportion of study participants with an unfavorable outcome were computed for each treatment arm. The odds ratio and its 95% confidence interval of favorable outcome between the experimental arm and each control arm were also calculated. The favorable versus unfavorable dichotomies for each of the 4 test instruments were also assessed. Again, no statistically significant differences in the proportion of patients having an unfavorable outcome were noted among the 3 treatment groups. Thus, we are reporting on pooled neurologic data for all 55 of the subjects from all 3 treatment cohorts who completed the clinical study.

### Neurological manifestations

#### General neurologic findings

The analyses in this report are focused on the subjects (n = 55) confirmed to have WNV disease by positive WNV IgM in serum and/or CSF. Neurological score at both baseline (day 0; n = 55) and those with a last follow-up at day 90 (n = 48) were assessed.

Overall, the predominant neurological impairments in these patients were coma, cognitive impairment, cranial neuropathy, tremor, and/or weakness, in multiple combinations (Table 2). The analyses focused on assessing these variables.

**Table 2:** Symptoms at initial and follow-up clinical presentation

**Initial evaluation (N = 55)**	**Normal**	**Coma**	**Cognitive impairment**	**Cranial neuropathy**	**Tremor**	**Weakness**
	4	14	25	9	19	27
						20 generalized
						4 asymmetric limb
						2 upper extremities
						1 paraparesis
**Follow-up Evaluation (N = 48)**	**Normal**	**Coma**	**Cognitive impairment**	**Cranial neuropathy**	**Tremor**	**Weakness**
	19	1	9	4	11	23
						6 generalized
						7 asymmetric limb
						2 upper extremities
						4 paraparesis
						4 hemiparesis

When an individual had multiple deficits, they are listed above multiple times under each deficit they possessed at that time.

The sensory and reflex examinations were found to be non-informative to the analysis and somewhat less consistent than the other findings. The measures of finger-to-nose and heel-to-shin ataxia were typically consistent and associated with measures of 3-4/5 weakness in the limbs noted to have ataxia. Thus, there were not reports of isolated ataxia without associated weakness, resulting in an inability to confidently report on ataxia.

The most frequent detected cranial neuropathies were manifested as facial weakness, and these were noted in the analysis for the presence/absence of impaired cranial nerve function. Intention tremors were uncommon and were typically associated with limb weakness. While there was no strong correlation between report of an intention tremor and the report of tremors in general, it cannot be safely assumed that these were resting tremors, as not all these patients were assessed by experienced neurologists. Thus, we will report on tremors, not otherwise further characterized.

#### Neurologic outcomes

Descriptive results of the following key variables and their patterns of impairment in the pre- and post-assessments are summarized in Table 2.

Table 2 defines the numbers of individuals both at the first evaluation (baseline) and follow-up who exhibited the defined neurological deficits, with those individuals exhibiting multiple deficits listed cumulatively. The patterns of weakness were subdivided for each individual. Overall, 93% of the patients presented with a neurological deficit, with 25% of the total cohort presenting with coma, and 45% with cognitive impairment, resulting in 70% of all patients presenting with some form of alteration in consciousness/cognitive function. In addition, 49% presented with some form of weakness, 35% with tremor, and 16% with cranial neuropathy.

Seven of the 55 WNV confirmed patients died during the study (mortality rate of 13%; 6 before day 90 evaluation and one after day 90). Five of those patients were in a coma upon entry into the study, and the other two had cognitive impairment and generalized weakness. Three of the patients who died succumbed to secondary respiratory complications, two died of cardiac complications, and two presented in a coma with flaccid quadriparesis requiring intubation and their families subsequently withdrew care. The median age at death was 71 years and all were Caucasian. Five of those patients were men and two were women.

Overall, most initially-evident deficits improved, except for weakness. However, the profile of the weakness in individuals changed such that, over time, fewer limbs were affected and the degree of strength impairment was reduced.

Tables 3 and 4 report the symptom profiles and numbers of individuals with these patterns of deficits for both the initial presentation (Table 3) and follow-up (Table 4).

**Table 3:** Initial clinical profiles (N = 55)

Normal	4
Coma	14
*Coma +*	*6 tremor*
	*1 dysconjugate gaze*
	*+ decreased gag*
	*7 isolation*
Just cognitive impairment	5
Just tremor	1
Just weak	6
*Generalized*	*3*
*Asymmetric limb paresis*	*2*
*Bilateral UEs*	*1*
Asymmetric limb paresis + facial weakness + dysconjugate gaze	1
General + facial weakness + dysconjugate gaze	1
Facial weakness + decreased gag reflex	1
Impaired cognition + tremor + general weakness	5
Impaired cognition + tremor + general weakness + dysconjugate gaze/pupil reactivity	1
Impaired cognition + tremor + asymmetric limb paresis	1
Impaired cognition + tremor + paraparesis	1
Impaired cognition + general weakness	9 (with 3 of these w/facial weakness)
Impaired cognition + UEs weak	1
Impaired cognition + tremor	2
Tremor + facial weakness + decreased gag reflex	1
Tremor + general weakness	1

UEs: upper extremities.

**Table 4:** Follow-up clinical profiles (N = 48)

Normal	19
Coma	1
Just cognitive impairment	1
Just tremor	3
Just weak	12
*General*	*3*
*Asymmetric limb paresis*	*2*
*Paraparesis*	*4*
*Bilateral UEs*	*1*
*Hemiparesis*	*2*
Asymmetric limb paresis + facial weakness + dysconjugate gaze	1
Asymmetric limb paresis + facial weakness	1
Asymmetric limb paresis + tremor	1
Impaired cognition + tremor + general weakness	2
Impaired cognition + tremor + facial weakness	1
Impaired cognition + general + facial weakness	1
Impaired cognition + tremor + hemiparesis	1
Impaired cognition + tremor + asymmetric limb paresis	2
Impaired cognition + hemiparesis	1
Tremor + UEs weak	1

UEs: upper extremities.

Considering the range of profound deficits noted across subjects, it was notable that four of the 55 (7%) subjects in the group were found to be normal at onset and all of these remained normal throughout the study. By the end of the study, another 14 had returned to normal in all domains (33% of those who started the study). Of those who returned to a normal baseline, only two were initially in a coma, three initially had an isolated cognitive impairment, one initially had an isolated tremor, 3 had general weakness, and 5 had multiple domains of deficits.

### Cross-sectional analyses of demographic characteristics

#### Gender

Thirty percent of the sample population were female and, at the initial evaluation, approximately one-third of them were in a coma. Only one of the four women (25%) who were comatose at presentation was not in a coma 90 days later. Notably, eight of the 10 males (80%) who were comatose at presentation were not in coma 90 days later.

#### Ethnicity

None of the African-Americans (N = 2) or Hispanics (N = 10) presented in a coma, whereas all of the Native Americans (N = 2) presented clinically in a coma (*p* = .01). Of the subjects who could cooperate for strength testing at long-term follow-up at 90 days (N = 48), Caucasians were over-represented in the generalized 4-limb weakness group, whereas Hispanics were over-represented in the 2-limb category and underrepresented in the 4-limb category (p = .01).

#### Age

The effect of age on weakness measured in those who could cooperate for testing at day 90 (N = 48) showed a trend whereby older patients were more likely to exhibit weakness. There was a trend toward an over-representation of weakness in the more severely impaired ranges (p = .08). Using age (by decade) as a predictor in a linear regression, one could reliably predict the degree of a weakness deficit (*p =* .01, *R*^2^ = .132). Actual age as the predictor in the linear regression also reliably predicted the degree of weakness deficit (*p* = .03, *R*^2^ = .101). There was no relationship between age and degree of weakness reported at the *onset* of the study (chi-squared and linear regression, *p* > .80).

### Longitudinal analyses of factors

We subtracted the Outcome Scores (range of 0–6 on mortality and main neurological outcome factors) from those at the initial evaluation. These scores were assessed across all neurologic factors using chi-square statistics. Ethnicity did not show the expected distribution related to resolution of coma, *p* = .001. At the onset of the study, 11 of the 40 Caucasian, both of the 2 Native American, and 1 of the ‘Other” ethnicity subjects were in a coma. None of the 10 Hispanic or 2 African-American subjects was in a coma at the onset of the study. At follow-up evaluation, only 1 (Caucasian) of the 14 (N = 55 at beginning of study) patients remained in a coma, 8 recovered from coma, and 5 were dead (all Caucasian).

Assessing the change scores for the coma condition, those who were in a coma at presentation, but not at Day 90, were more likely to have a cognitive deficit at the termination of the study (*p* = .01).

As noted above, we calculated an Outcome Score from 0 (no coma, no cognitive deficits, no tremor, no cranial nerve problems, no weakness) to 5, with death being a score of 6 (N = 55). Age decade reliably predicted this Outcome Score (*p* = .04). Initial presentation in a coma also was a significant factor in predicting outcome (*p* < .0001). Those in a coma at presentation were more likely to have a cognitive deficit at the end of the study (*p* = .01).

### Regression analysis of outcome scores

A logistic regression initially yielded no significant results, so the presentation values were changed to continuous variables and a multiple regression analysis was performed to predict the Outcome Score. Stepwise regression revealed that only coma at Day 1 and cranial nerve pathology at Day 1 made unique contributions to the model, accounting for 33.2% of the variance in Outcome Score (*p <* .0001). Coma at Day 1 reliably predicted the Outcome Score when used as the sole regressor, *R*^2^ = .278, *p <* .0001. The model was significantly improved by adding cranial nerve pathology at Day 1 as a predictor, *R*^2^ = .332, *p = .*0463. Cognitive deficit at Day 1 trended toward significantly improving the model (*R*^2^ change .038, *p =* .084). None of the other predictors significantly improved the model (*p >* .25).

## Discussion

This study reports neurologic outcome data derived from a multicenter therapeutic trial involving patients with neuroinvasive WNV disease. The wide spectrum of neurologic impairments in these patients was similar to what has been reported in previous studies. The patients presented with a combination of deficits that included coma, mental status, cranial neuropathy, tremor, and/or weakness (including asymmetric limb weakness). This represents one of the largest studies providing prospectively acquired neurological outcomes data among American patients with WNV CNS disease.

The initial presentation of patients with WNV encephalitis and/or myelitis in this study demonstrated several salient characteristics of the neurological profile associated with this disease. Across all patients (n = 55), 93% presented with a significant neurological deficit detectable by clinical examination, with 49% having some form of weakness, 35% with tremor, and 16% with cranial neuropathy. Also included in the initial clinical profiles were five patients with isolated cognitive impairment as measured by the 3MS, one with tremor only, and six with weakness only, including two with single limb and one with bilateral arm weakness. Presentation with just these isolated findings not only points out the selective clinical features with which this viral infection can manifest, but also provides insight into the brain structures the virus may affect. To account for the cranial neuropathies and also for coma, brain stem involvement is implicated. Pathological processes affecting the basal ganglia and/or thalamus can result in cognitive impairments, tremors, coma, and/or generalized weakness. Asymmetric limb weakness has been classically associated with infection by polio virus, a picornavirus that affects the anterior horn cells of the spinal cord [28]. WNV plausibly affects the same cell populations in those patients who exhibit a similar pattern of neurological deficits.

Seven patients died after initial enrollment in the study. The number of deaths is too small to provide a clear pattern of those who are at most risk, but it is clear that presenting in a coma early was common in this selected population (5 of the 7). However, 14 patients presented in a coma, so not all who present in a coma have such a poor prognosis. In our study, these patients tend to be older in general (mean age at death approximately 65 years) than other subjects, Caucasian (all), and male (5 of 7 patients); however, there are individuals with similar profiles who experience some degree of recovery.

Despite the lack of specific treatment of proven value for WNV neuroinvasive disease, considerable natural recovery occurs. Overall, there were 14 comatose subjects at baseline evaluation, but only one at follow-up. The number of patients who exhibited a cognitive deficit fell from 25 at initial evaluation to 9 at their last evaluation. Cranial neuropathy was present in 9 at onset and only 4 patients at study conclusion. Of the 19 who initially had a tremor at enrollment, 11 continued to exhibit a tremor at follow-up.

Previous reports have described asymmetric limb paresis (similar to those reported with poliomyelitis), generalized weakness, paraparesis, and hemiparesis. We observed considerable evolution in the type as well as numbers with weakness. Some aspects of these changes were likely accounted for by resolution of impaired cognition, including coma, which allowed for a more complete and reliable evaluation of limb strength among other clinical findings. Whereas at onset there were 20 patients with generalized weakness and 4 with asymmetric limb weakness, 1 paraparesis, 2 upper extremity weakness, and none with hemiparesis, there were considerably more individuals with clearly defined, focal weakness (6 generalized weakness, 7 asymmetric limb weakness, 2 with UE weakness, 4 paraparesis, and 4 hemiparesis) at follow-up.

There are several factors that predict either severity of a patient’s initial deficits or probability of long-term recovery. While ethnicity appeared to be associated with patients’ deficits, interpretation of the findings is limited by the small numbers of patients in each group. Males more often resolved from an initial state of coma than did women. Initial presentation in a coma also was a significant factor in predicting outcome. In fact, initial presentation in a coma and cranial nerve deficits best predict the overall neurological outcome score at three months after enrollment. These same factors also predict whether a patient will have a cognitive deficit at follow-up. Coma and cranial nerve deficits in combination not only suggest significant neurological impairment, but also suggest the effect of the virus on the brainstem (or possibly additional thalamic involvement), which appears to predispose an individual to a poorer long-term outcome. Age is also a predictive factor for weakness at follow-up.

Several limitations in this study are worth noting. One of the key limitations is that the examinations were performed by different examiners, most of whom were internists/infectious diseases specialists, not neurologists. Another drawback to the study is the relatively small number of subjects, even though this is among the largest cohorts of prospectively followed patients reported to date. Given the unpredictable nature of the WNV outbreaks, the present study design was utilized to capture the largest number of patients for initial and follow-up evaluations, but subject recruitment was still very challenging. Study modifications that allow for more rapid and flexible subject recruitment (including use of a universal Institutional Review Board) in the earliest stages of an outbreak would likely increase the number of patients enrolled and expand the type of investigative tools that could be utilized (e.g., neuroimaging, EMG, etc.).

Prognostic determinations in previous studies have not included formal neurological evaluation or use of the 3MS administered to subjects to assess functional status. Prognosis with measures such as the Short Form 36 [29] have reported a ‘favorable’ outcome in most patients after one year in the typical patient, providing an estimate of when recovery asymptotes [30]. In contrast, a telephone follow-up of symptom presence and severity of neurological status suggested only 37% achieved a full recovery at 1 year, with older age being a significant negative predictor [25]. Another survey of 214 patients 7 months after their acute illness found that age and gender were not associated with persistent symptoms [31], which is contrast to our findings. Self-reported symptoms in conjunction with neurological assessment approximately a year after the acute illness revealed a variety of somatic, neurological, and cognitive complaints with 10 of 49 reporting tremor and one patient with asymmetric paralysis [24]. In the present study, there was a much wider array of neurological deficits detected at long-term follow-up examination and a higher percentage of individuals with deficits, arguing for formal neurological evaluations to most accurately assess neurological outcomes.

Given the limitations in this study, we chose the neurological measures in our analysis by reviewing the measures collected and finding the most consistent data that could be confirmed by converging scores from other measures (e.g., strength measures for limbs supported by multiple strength measures for muscle groups). Thus, limb strength is reported as an overall measure, which is not as precise as desirable. These WNV outbreaks do not produce a purely random sample, and the examinations also lacked corroborative imaging and/or electrophysiological studies due to study and site limitations. Finally, we collapsed 3 cohorts from a treatment trial to report this ‘natural history’ study since the treatment was found to be ineffective. These caveats notwithstanding, the present study provides one of the largest cross-sectional and longitudinal characterizations of the neurological deficits encountered in subjects with WNV neuroinvasive disease.

## Conclusion

This study represents one of the largest clinical investigations providing prospectively- acquired neurological outcomes data among American patients with WNV CNS disease. At initial clinical presentation (n = 55), 93% presented with a significant neurological deficit detectable by clinical examination, with 49% having some form of weakness, 35% with tremor, and 16% with cranial neuropathy. Also included in the initial clinical profiles were five patients with isolated cognitive impairment as measured by the 3MS, one with tremor only, and six with weakness only, including two with single limb and one with bilateral arm weakness. Seven patients died after initial enrollment in the study, with presentation in a coma being common in this subgroup (5 of the 7). In terms of long-term follow-up, 14 subjects presented in a coma at baseline evaluation, while only one of the survivors remained in a coma at final follow-up. The number of patients who exhibited a cognitive deficit fell from 25 at initial evaluation to 9 at their last evaluation. Cranial neuropathy was present in 9 at onset and only 4 patients at study conclusion. Of the 19 who initially had a tremor at enrollment, 11 continued to exhibit a tremor at follow-up.

## Competing interests

The authors declare that they have no competing interests.

## Authors’ contributions

JH Jr. designed the study, analyzed the data, drafted the manuscript. GT, MAK, H-SC, and JFS analyzed the data and edited the manuscript. YL oversaw the statistical analyses. AGA designed the study and oversaw the data acquisition. PJ oversaw the data acquisition. JWG Jr. and RJW designed the study, oversaw the data acquisition, analyzed the data, and edited the manuscript. All authors read and approved the final manuscript.

## Pre-publication history

The pre-publication history for this paper can be accessed here:

http://www.biomedcentral.com/1471-2334/14/248/prepub
